# Verification of nucleotide sequence reagent identities in original publications in high impact factor cancer research journals

**DOI:** 10.1007/s00210-023-02846-2

**Published:** 2024-01-09

**Authors:** Pranujan Pathmendra, Yasunori Park, Francisco J. Enguita, Jennifer A. Byrne

**Affiliations:** 1 School of Medical Sciences, Faculty of Medicine and Health, The University of Sydney, Camperdown, NSW 2050 Australia; 2 Instituto de Medicina Molecular João Lobo Antunes, Faculdade de Medicina, Universidade de Lisboa, Av. Prof. Egas Moniz, 1649-028 Lisbon, Portugal; 3 NSW Health Statewide Biobank, NSW Health Pathology, Camperdown, NSW 2050 Australia

**Keywords:** circRNA, Error, Gene research, Nucleotide sequence

## Abstract

Human gene research studies that describe wrongly identified nucleotide sequence reagents have been mostly identified in journals of low to moderate impact factor, where unreliable findings could be considered to have limited influence on future research. This study examined whether papers describing wrongly identified nucleotide sequences are also published in high-impact-factor cancer research journals. We manually verified nucleotide sequence identities in original *Molecular Cancer* articles published in 2014, 2016, 2018, and 2020, including nucleotide sequence reagents that were claimed to target circRNAs. Using keywords identified in some 2018 and 2020 *Molecular Cancer* papers, we also verified nucleotide sequence identities in 2020 *Oncogene* papers that studied miRNA(s) and/or circRNA(s). Overall, 3.8% (251/6647) and 4.0% (47/1165) nucleotide sequences that were verified in *Molecular Cancer* and *Oncogene* papers, respectively, were found to be wrongly identified. Wrongly identified nucleotide sequences were distributed across 18% (91/500) original *Molecular Cancer* papers, including 38% (31/82) *Molecular Cancer* papers from 2020, and 40% (21/52) selected *Oncogene* papers from 2020. Original papers with wrongly identified nucleotide sequences were therefore unexpectedly frequent in two high-impact-factor cancer research journals, highlighting the risks of employing journal impact factors or citations as proxies for research quality.

**Supplementary Information:**

The online version contains supplementary material available at 10.1007/s00210-023-02846-2.

## Introduction

Despite technological advances, growing research workforce capacity, and billion-dollar budgets devoted to biomedical research in first-world countries, biomedical research translation continues to fall short of the expectations generated by research investments (Bowen and Casadevall 2015). Inefficient research translation is fueled by the reproducibility crisis, where many pre-clinical research results cannot be independently reproduced (Mobley et al. 2013; Pusztai et al. 2013; Errington et al. 2021). The emphasis upon publication of positive findings has likely led to publication of false-positive results (Pusztai et al. 2013; Smaldino and McElreath 2016; Kaelin 2017). Where these results are not reproduced by other studies, these contradictory or discordant results may be less likely to be reported, leading to a growing problem of falsely positive research results in the biomedical literature (Smaldino and McElreath 2016; Kaelin 2017).

While most incorrect pre-clinical research is believed to derive from genuine research (Brown et al. 2018), some irreproducible research results may reflect data falsification and fabrication (Stroebe et al. 2012; Gopalakrishna et al. 2022). Over the past several years, the analysis of research fraud has shifted from focusing on research fraud perpetrated by individuals, to include research fraud that may be enabled by organizations known as paper mills (Byrne 2019; Byrne and Christopher 2020; COPE, STM 2022; Christopher 2021; Heck et al. 2021; Parker et al. 2022; Bricker-Anthony and Giangrande 2022; Frederickson and Herzog 2022). There is growing evidence suggesting that human genes could be targeted by paper mills for the production of preclinical research manuscripts (Byrne and Labbé 2017; Qi et al. 2017; Han and Li 2018; Byrne et al. 2019, 2021b, 2022; Labbé et al. 2019; Clark and Buckmaster 2021; Cooper and Han 2021; Seifert 2021; Park et al. 2022; Pérez-Neri et al. 2022; Wittau et al. 2023). The rapid production of many gene research manuscripts at minimal cost could provide limited time for quality control, which could result in errors such as wrongly identified nucleotide sequence reagents (Byrne and Labbé 2017; Byrne et al. 2019).

Wrongly identified RT-PCR primers and gene knockdown reagents could arise in different research contexts, as the identities of these reagents typically cannot be judged by eye (Byrne et al. 2019, 2021b) (Table 1). As the disclosure of short nucleotide sequences also enables their reuse in future studies, the semi-automated tool Seek & Blastn was created to verify the identities of published nucleotide sequence reagents that are claimed to target human genes and transcripts (Labbé et al. 2019). The application of Seek & Blastn has demonstrated the widespread occurrence of wrongly identified nucleotide sequence reagents in repetitive human gene research papers (Labbé et al. 2019; Byrne et al. 2021b; Park et al. 2022). Our most recent application of Seek & Blastn screened over 11,700 original human research papers and identified 712 papers that described wrongly identified nucleotide sequence(s), including papers that studied gene functions in the context of chemosensitivity or -resistance (Park et al. 2022). Seek & Blastn screening of original papers in the journals *Gene* and *Oncology Reports* revealed that yearly proportions of original papers with wrongly identified sequence(s) ranged from 0.5 to 4.2% and 8.3 to 12.6%, respectively (Park et al. 2022).

**Table 1:** Potential causes of wrongly identified nucleotide sequence reagents, possible predisposing factors, and how errors can be detected

Cause	Predisposing factors	Detection
• Reagents designed based on incorrect sequence information and/or metadata (Goudey et al. 2022)	• Recently identified or understudied genes, transcripts, genomic sequences	• Full disclosure of how all nucleotide sequence reagents were designed • Unexpected results for positive/negative controls • Publication of negative or contradictory results (Park et al. 2022)
• Reagent design informed by limited understanding of methodology (e.g., specific reagent targeting requirements)	• Inexperienced researchers • Poor experimental design • Limited supervision or mentoring	
• Errors introduced during manuscript preparation, e.g., cut and paste errors	• Studies involving many individual experiments and/or genetic targets • Studies involving experiments that target similarly named genes (i.e., different members of multi-gene families)	• Check all reagent identities before publication or experimental use (Byrne et al. 2022) • Publication of negative or contradictory results (Park et al. 2022)
• Wrongly identified nucleotide sequence reagents repurposed from previous studies	• Wrongly identified reagents described in many publications (e.g., incorrect non-targeting gene knockdown controls) (Byrne and Labbé, 2017; Byrne et al. 2021b)	

Most human gene research papers with wrongly identified nucleotide sequences have been identified in journals of low to moderate impact factor (IF) (Byrne and Labbé 2017; Labbé et al. 2019; Byrne et al. 2021b; Park et al. 2022). This finding is likely to at least partly reflect the skewed distribution of journal IF’s (Romanovsky 2019; Siler and Larivière 2022), where high IF cancer research journals defined by an IF ≥ 7.0 (Kempf et al. 2018) correspond to ~ 20% of cancer research journals. While recognizing the limited utility of journal IF as a measure of research quality (Siler and Larivière 2022), the perceived significance of human gene research papers with wrongly identified sequences could be discounted through their publication in lower IF journals. Our team has also described examples of human gene research papers with wrongly identified nucleotide sequences that were published in high IF journals (Labbé et al. 2019; Park et al. 2022). It is currently unclear whether low numbers of human gene research papers with wrongly identified nucleotide sequences in high IF journals simply reflect low numbers of high IF journals (Romanovsky 2019; Siler and Larivière 2022), and/or that few papers with wrongly identified nucleotide sequences have been published by high IF journals.

**Fig. 1:**
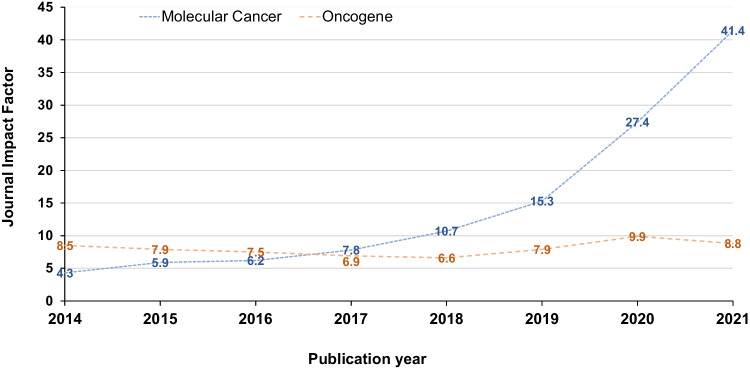
Journal impact factors (https://clarivate.com/) (*Y*-axis) for *Molecular Cancer* (blue) and *Oncogene* (orange) from 2014 to 2021 (*X*-axis). Journal impact factors have been rounded to one decimal place

We have therefore undertaken a literature screening approach to examine the frequency of human gene research papers with wrongly identified nucleotide sequence reagents in two high IF cancer research journals, as judged by 2019 journal IF (https://clarivate.com/). We chose to examine *Molecular Cancer*, an online, open-access journal published by BMC (Springer Nature), as Seek & Blastn screening of keyword-driven literature corpora had previously identified *Molecular Cancer* papers with wrongly identified nucleotide sequences that were published in 2014 (Park et al. 2022). Although *Molecular Cancer* was not a high IF journal in 2014 (IF = 4.3), *Molecular Cancer* has experienced a marked rise in journal IF, reaching IFs of 15.3 in 2019, 27.4 in 2020, and 41.4 in 2021 (Fig. 1). As a result, *Molecular Cancer* was the 3rd-ranked molecular biology and biochemistry journal in 2020 and 2021, following only *Nature Medicine* and *Cell*. We also verified nucleotide sequence reagent identities in a selected corpus of 2020 *Oncogene* papers. *Oncogene* is published by Springer Nature under a hybrid open-access/subscription publication model. Unlike *Molecular Cancer*, *Oncogene* has shown a relatively stable journal IF ranging from 6.6 to 9.9 during 2014–2021 (Fig. 1).

As most *Molecular Cancer* papers described nucleotide sequence reagents in supplementary files and not in the publication text, these papers proved to be unsuitable for Seek & Blastn screening (Labbé et al. 2019). We therefore manually verified the identities of all nucleotide sequence reagents that were claimed to target unmodified (wild-type) human gene targets in original *Molecular Cancer* papers published in 2014, 2016, 2018, and 2020. These publication years were chosen so that proportions of *Molecular Cancer* papers could be compared with those previously identified in *Gene* and *Oncology Reports* in 2014, 2016, and 2018 (Park et al. 2022). As some *Molecular Cancer* papers described nucleotide sequence reagents that were claimed to target human circular RNA (circRNA) transcripts, we developed protocols to verify the identities of circRNA targeting reagents. Using keywords identified in some *Molecular Cancer* papers (miRNA, miR, circular RNA, or circRNA), we undertook keyword-driven searches of all original 2020 *Oncogene* papers. We manually verified the identities of all nucleotide sequence reagents that were claimed to target unmodified human gene targets in all 2020 *Oncogene* papers that referred to microRNAs and/or circRNAs.

As we will describe, these analyses identified unexpectedly high proportions of human gene research papers with wrongly identified nucleotide sequences in two high IF cancer research journals. Our results therefore indicate that human gene research publications that describe wrongly identified nucleotide sequences may be unexpectedly frequent in some high IF cancer research journals.

## Methods

### Identification of literature corpora

*Molecular Cancer* papers were retrieved via the Web of Science using the search criteria: PY = “2014, 2016, 2018, 2020,” SO = “MOLECULAR CANCER,” AND DT = “Article.” Article titles were used as search queries on the *Molecular Cancer* website to obtain pdfs and supplementary files. Based on features of some *Molecular Cancer* papers with wrongly identified nucleotide sequence(s), selected *Oncogene* papers were retrieved via the Web of Science using the search criteria: PY = “2020,” SO = “ONCOGENE,” DT = “Article,” and keywords = [(“Circular RNA*.mp.” OR “circRNA*.mp.”) OR (“microRNA*.mp. OR “miR*.mp.”)]. *Oncogene* article titles were used as search queries to obtain article pdfs and supplementary files through the University of Sydney library.

### Visual inspection of articles

Each article was subjected to visual screening and considered eligible for analysis if the study described the sequence of at least one nucleotide sequence reagent that was claimed to target an unmodified (wild-type) human transcript or genomic region. Publications including supplementary files were visually inspected to determine the claimed genetic and/or experimental identity of each nucleotide sequence. If the claimed target or experimental use of any sequence was not evident, or if a sequence was claimed to target a species other than human, the sequence was excluded from further analysis. We included papers with post-publication notices such as retractions and published corrections, except where post-publication corrections had corrected all wrongly identified nucleotide sequences at the time of publication screening. Eligible papers were identified by their PMIDs. Nucleotide sequences and their claimed identities were manually extracted from text and/or supplementary files using copy/paste functions, or transcribed from figures, and recorded in Microsoft Excel.

### Manual verification of nucleotide sequence reagent identities

Nucleotide sequence reagents that were claimed to target human protein-coding genes and microRNAs were analyzed as described (Byrne et al. 2021a; Park et al. 2022). GeneCards (Stelzer et al. 2016) and GenBank (Sayers et al. 2019) were used to clarify synonymous human gene identifiers. For nucleotide sequence reagents that were claimed to target long non-coding RNAs (lncRNAs), the claimed identifier was searched on lncBASE (Karagkouni et al. 2020) and GeneCards (Stelzer et al. 2016) to identify the genomic coordinates of the claimed lncRNA. Claimed targeting reagent sequences were queried using BLAT against the GRCh38/hg38 assembly (Lee et al. 2022) and Blastn (Altschul et al. 1990) as described (Park et al. 2022).

Nucleotide sequence reagents that were claimed to target genomic sequences including gene promoters were queried using BLAT against the GRCh38/hg38 assembly (Lee et al. 2022) as described (Park et al. 2022). Claimed gene promoter targeting reagents were accepted as targeting if these reagents mapped within 100-kb upstream of the claimed target gene and if reagents did not include coding gene exons. Where the claimed reagent identity did not match the verified identity, sequences were queried using BLAT against earlier human genome assemblies (Lee et al. 2022).

### Manual verification of claimed circular RNA targeting reagents

#### Verification of RT-PCR primers claimed to target circRNAs

circRNAs are alternatively spliced transcripts where gene exons are joined through back-splicing to create circular transcripts (Dudekula et al. 2016; Zhong et al. 2018; Nielsen et al. 2022). RT-PCR amplification of circRNAs requires two sets of RT-PCR primers (Dudekula et al. 2016; Zhong et al. 2018, 2019; Nielsen et al. 2022). Divergent RT-PCR primers are used to amplify the claimed circRNA by facing towards and amplifying across the circRNA BSJ (Dudekula et al. 2016; Zhong et al. 2018, 2019; Nielsen et al. 2022). Divergent RT-PCR primers should therefore not amplify linear transcripts from the host or any other human gene. In contrast, convergent RT-PCR primers are employed to amplify linear transcripts, typically from the claimed host gene (Dudekula et al. 2016; Zhong et al. 2018, 2019; Nielsen et al. 2022).

**Fig. 2:**
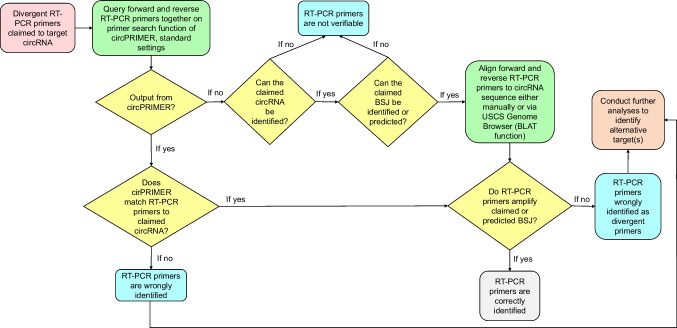
Flow chart summarizing the workflow that was used to manually verify the identities of divergent RT-PCR primers claimed to target human circRNAs

For claimed divergent RT-PCR primers, forward and reverse primers were first queried on circPRIMER (Zhong et al. 2018) using standard settings (Fig. 2). RT-PCR primers were accepted as correctly targeting if circPRIMER aligned both RT-PCR primer sequences to the claimed circRNA(s), such that RT-PCR primers faced towards and were predicted to amplify the back splice junction (BSJ) (Fig. 2). If circPRIMER analyses produced no output, we then checked whether the claimed circRNA was indexed by a publicly available circRNA database such as circBASE (Glažar et al. 2014) or circATLAS (Wu et al. 2020) through the disclosure of a specific circRNA identifier, or if the circRNA sequence and/or its genomic sequence coordinates were disclosed by the authors. If the claimed circRNA could not be identified, the claimed divergent RT-PCR primers were classified as non-verifiable (Patop and Kadener 2018). If the claimed circRNA could be identified but the BSJ could not be identified or predicted, claimed divergent RT-PCR reagents were also classified as non-verifiable.

If the claimed BSJ sequence was either disclosed or the associated genomic coordinates could be predicted, divergent RT-PCR primers were then queried either using the BLAT function of circBASE (Glažar et al. 2014), manually mapped to the claimed circRNA sequence, and/or queried using BLAT against the GRCh38/hg38 genomic assembly (Lee et al. 2022). Claimed divergent RT-PCR primers were classified as wrongly identified if they did not amplify the (predicted) BSJ (Fig. 2). Wrongly identified RT-PCR primers were subjected to further analyses to classify these reagents according to nucleotide sequence error categories (see below), as described (Park et al. 2022). Claimed convergent RT-PCR primers were verified as previously described for RT-PCR primers targeting linear transcripts (Labbé et al. 2019; Byrne et al. 2021a, 2021b; Park et al. 2022).

### Verification of single-nucleotide sequence reagents claimed to target circRNAs

**Fig. 3:**
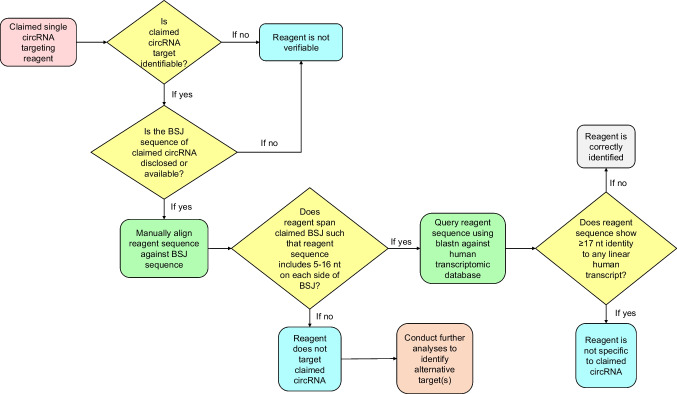
Flow chart summarizing the workflow that was used to manually verify the identities of single-nucleotide sequence reagents (siRNAs, shRNAs, other oligonucleotide probes) claimed to target human circRNAs

Single reagents such as si/shRNAs and other oligonucleotides acquire circRNA specificity by targeting specific BSJ sequences (Dudekula et al. 2016; Nielsen et al. 2022). We first determined whether the claimed circRNA was indexed in a publicly available circRNA database, as described above, and whether the BSJ sequence could be identified (Fig. 3). If claimed circRNA or the BSJ sequence could not be identified, reagents were classified as non-verifiable (Fig. 3).

Verifiable single reagents were manually aligned against the claimed circRNA BSJ sequence (Fig. 3). Single reagents were classified as correctly targeting if they showed 100% identity to 5–16 nucleotides on each side of the BSJ (Dudekula et al. 2016). If a claimed circRNA targeting reagent showed 100% identity to 17 or more consecutive nucleotides of any human linear transcript, including transcripts from the claimed host gene, the reagent was classified as wrongly identified, as such reagents would not be predicted to discriminate between circular and linear transcripts.

### Classification of wrongly identified reagents according to error categories

Wrongly identified nucleotide sequence reagents were classified according to previously described error categories, namely (i) claimed targeting reagents that were predicted to target another human gene or genomic sequence, (ii) claimed targeting reagents that were predicted to be non-targeting in human, and (iii) claimed non-targeting reagents that were predicted to target a human gene or transcript (Labbé et al. 2019; Byrne et al. 2021b; Park et al. 2022). Claimed circRNA targeting reagents (divergent RT-PCR primers, si/shRNAs, molecular probes) that were predicted to (also) target linear transcripts (including from the claimed host gene) were classified as targeting a different gene/transcript from that claimed (category (i) above).

### Summary of how nucleotide sequence reagent identities were manually verified

This study was conducted in the context of a student project (by PP), and hence all nucleotide sequence identities were verified by PP as described above. YP supported nucleotide sequence reagent identity verification in the early project stage, to ensure methodological consistency (Park et al. 2022). PP and JAB met regularly to discuss identity verification results for individual nucleotide sequences. JAB visually inspected the summary results for all nucleotide sequences that were predicted to be wrongly identified and recommended individual results for rechecking by PP and/or JAB. PP and JAB consulted with FJE for advice on targeting parameters and workflows for claimed divergent RT-PCR primers and single-nucleotide sequence reagents that were claimed to specifically target circRNAs (Figs. 2 and 3). JAB manually verified alignments between single circRNA reagents and claimed BSJ sequences for all single circRNA reagents that were predicted to not target the claimed BSJ. PP then rechecked the identities of all wrongly identified nucleotide sequences prior to reporting.

### Additional publication analyses

For each eligible article, we recorded the number and proportion of wrongly identified nucleotide sequence reagents. We also recorded the numbers and identities of non-verifiable circRNA reagents, noting that we did not categorize non-verifiable reagents as wrongly identified. Publications were flagged if they included at least one wrongly identified nucleotide sequence reagent. Papers that described non-verifiable circRNA targeting reagent(s) but no wrongly identified nucleotide sequences were reported separately. Proportions of papers with wrongly identified sequence(s)/papers analyzed and papers with wrongly identified sequence(s)/papers screened and wrongly identified nucleotide sequences/nucleotide sequences analyzed were calculated for journals and publication years using MS Excel.

Publication titles were visually inspected to identify human gene or transcript identifiers, human cancer types, and drug identifiers which were confirmed through Google searches. Human genes were categorized as either protein-coding or ncRNAs according to GeneCards (Stelzer et al. 2016). The country of origin and institutional affiliation were identified as described (Park et al. 2022). Where there was no numeric majority, the first author’s affiliation was used to decide the country of origin and/or institutional affiliation. PubPeer notifications (Barbour and Stell 2020) were identified on 16 January 2023. Reported numbers of post-publication notices are those identified through PubMed and Google Scholar searches conducted on 17 January 2023. Citations according to Google Scholar were collected on 22 January 2023.

### Statistics analyses

Fisher’s exact tests conducted on GraphPad PRISM compared proportions of *Molecular Cancer* papers according to publication year, and countries and institutions of origin. Shapiro-Wilk’s test was used to test for normality. The Mann-Whitney test was conducted to compare median numbers of wrongly identified sequences per *Molecular Cancer* article according to publication year, where reported p values have not been corrected for multiple comparisons. For all *Molecular Cancer* papers with wrongly identified nucleotide sequence(s), Spearman’s rank correlation coefficient was calculated between the numbers of wrongly identified sequences and numbers of analyzed nucleotide sequences per article. Graphs were produced on GraphPad PRISM 9.2.

## Results

### *Molecular Cancer* corpus

**Fig. 4:**
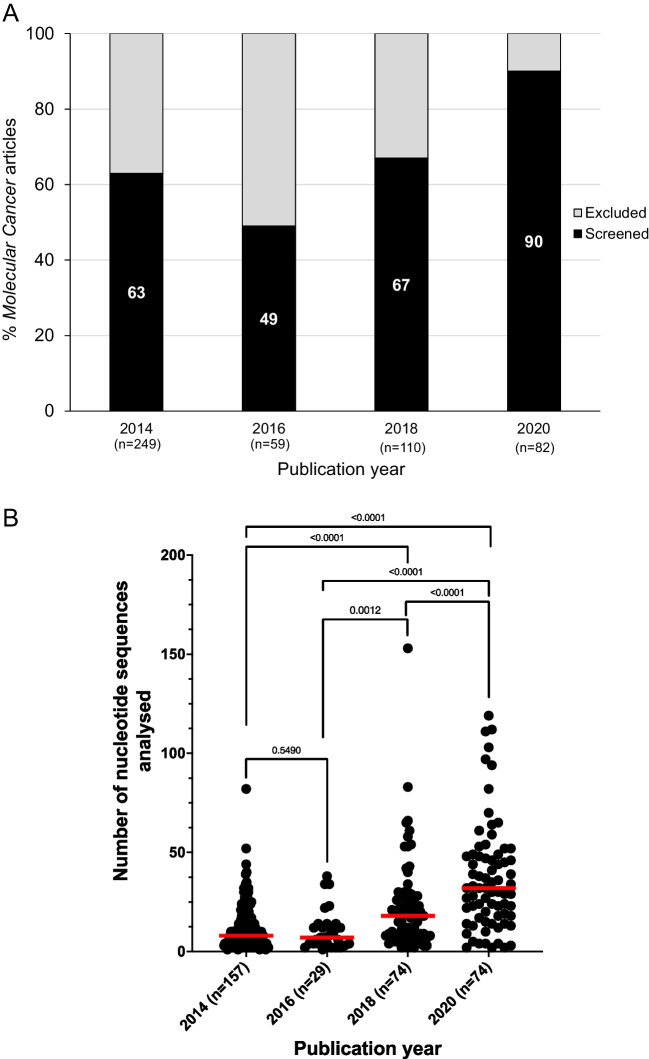
Summary of original papers published in *Molecular Cancer* in 2014, 2016, 2018, and 2020. Numbers of original *Molecular Cancer* papers (analyzed) per year are shown below the *X*-axis. **A** Percentages of original *Molecular Cancer* papers (*Y*-axis) that were either screened (black, percentage values shown in white text) or excluded from analysis (gray) per year (*X*-axis). **B** Numbers of nucleotide sequences per *Molecular Cancer* paper (*Y*-axis) according to publication year (*X*-axis). Only original *Molecular Cancer* papers that described at least one nucleotide sequence reagent were included in these analyses. Individual/median numbers of nucleotide sequences/paper are shown as black dots/red horizontal lines, respectively. The Mann-Whitney test was employed to compare median nucleotide sequence numbers/paper according to publication year, as indicated by *p* values

In total, 500 original *Molecular Cancer* papers were published in 2014, 2016, 2018, and 2020 (Table 2), where numbers of original papers ranged from 59 papers in 2016, to 249 papers in 2014 (Fig. 4A). Most (334/500, 67%) original *Molecular Cancer* papers were included for analysis as they described human research and included at least one nucleotide sequence that was claimed to target a non-modified human gene or genomic sequence (Fig. 4A, Table 2). The proportions of *Molecular Cancer* papers that met the study inclusion criteria ranged from 29/59 (49%) in 2016 to 74/82 (90%) in 2020 (Fig. 4A).

**Table 2:** *Molecular Cancer* and *Oncogene* corpora that were screened for wrongly identified nucleotide sequence reagents

	*Molecular Cancer*	*Oncogene*
Corpus description	All original papers published in 2014, 2016, 2018, and 2020	Keyword driven search of original papers published in 2020
Number of original papers screened	500	52
Proportion (percentage) of all original papers that were eligible for analysis	334/500 (67%)	42/52 (81%)
Total number of nucleotide sequence reagents eligible for fact-checking	6647	1165
Number of nucleotide sequence reagents per article, median (range)	13 (1–153)	20 (2–115)
Proportion (percentage) of wrongly identified nucleotide sequence reagents	251/6647 (3.8%)	47/1165 (4.3%)
Percentage (proportion) of problematic papers	91/500 (18%)	21/52 (40%)
Number of wrongly identified nucleotide sequences per article, median (range)	2 (1–14)	2 (1–5)
Proportion (percentage) of wrongly identified nucleotide sequence reagents according to error types:	251/251 (100%)	47/47 (100%)
Claimed targeting reagents that were predicted to target a different human gene or genomic sequences	135/251 (54%)	24/47 (51%)
Claimed targeting reagents that were predicted to be non-targeting in human	114/251 (45%)	23/47 (49%)
Claimed non-targeting reagents that were predicted to target a human gene	2/251 (0.8%)	0/47 (0%)

The 334 *Molecular Cancer* papers included 6647 nucleotide sequences, with a median of 13 nucleotide sequences/paper (range 1–153) (Table 2). The numbers of nucleotide sequence reagents per paper progressively increased from 2014 to 2020 (Fig. 4B). For example, the median number of nucleotide sequences per paper increased from 8 sequences/paper in 2014, to 32 sequences/paper in 2020 (Mann-Whitney test, *p* < 0.0001, *n* = 231) (Fig. 4B).

Whereas no 2014 or 2016 *Molecular Cancer* papers described nucleotide sequences that were claimed to target human circular RNAs (circRNAs), 39 *Molecular Cancer* papers in 2018 and 2020 described circRNA targeting reagents. As we had not previously verified the identities of circRNA targeting reagents, new protocols were developed to recognize the particular targeting requirements of some circRNA reagents (Figs. 2 and 3, see the “Methods” section).

### *Molecular Cancer* papers with wrongly identified nucleotide sequence(s)

**Fig. 5:**
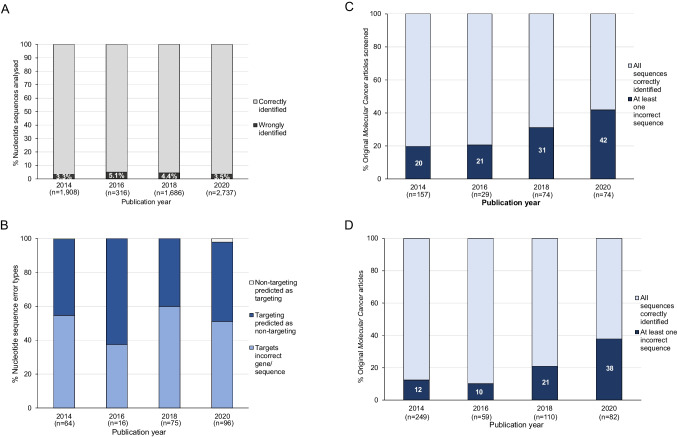
Summary of original *Molecular Cancer* papers in 2014, 2016, 2018, and 2020 that described at least one wrongly identified nucleotide sequence. **A** Percentages of nucleotide sequences (*Y*-axis, log scale) that were correctly (light gray) or wrongly identified (dark gray, percentages shown in white text) per publication year (*X*-axis). Numbers of nucleotide sequences analyzed in *Molecular Cancer* papers per year are shown below the *X*-axis. **B** Percentages of wrongly identified nucleotide sequences according to nucleotide sequence identity error types (*Y*-axis) and publication year (*X*-axis). Nucleotide sequence identity error types are shown as follows: claimed targeting reagents predicted to target a different gene or sequence (mid blue); claimed targeting reagents predicted to be non-targeting in human (dark blue); claimed non-targeting reagents predicted to target a human gene (light gray). Numbers of wrongly identified nucleotide sequences per publication year are shown below the *X*-axis. **C**, **D** Percentages of screened (**C**) or original *Molecular Cancer* papers (**D**) (*Y*-axes) that described at least one wrongly identified reagent (dark blue, percentages shown in white text) versus all other papers (light blue), according to publication year (*X*-axis). Numbers of papers per year are shown below the *X*-axis

Of the 6647 nucleotide sequences whose identities were manually verified, 251 (3.8%) nucleotide sequences were predicted to be wrongly identified (Table 2, Fig. 5A, Table S1). Similar proportions of incorrect sequences represented targeting reagents that were either verified to target a different human gene or genomic sequence (135/251, 54%), or predicted to be non-targeting in human (114/251, 45%) (Table 2, Fig. 5B). In contrast, very few (2/251, 0.8%) wrongly identified sequences represented claimed non-targeting si/shRNA reagents that were instead predicted to target a human gene (Table 2, Fig. 5B).

**Fig. 6:**
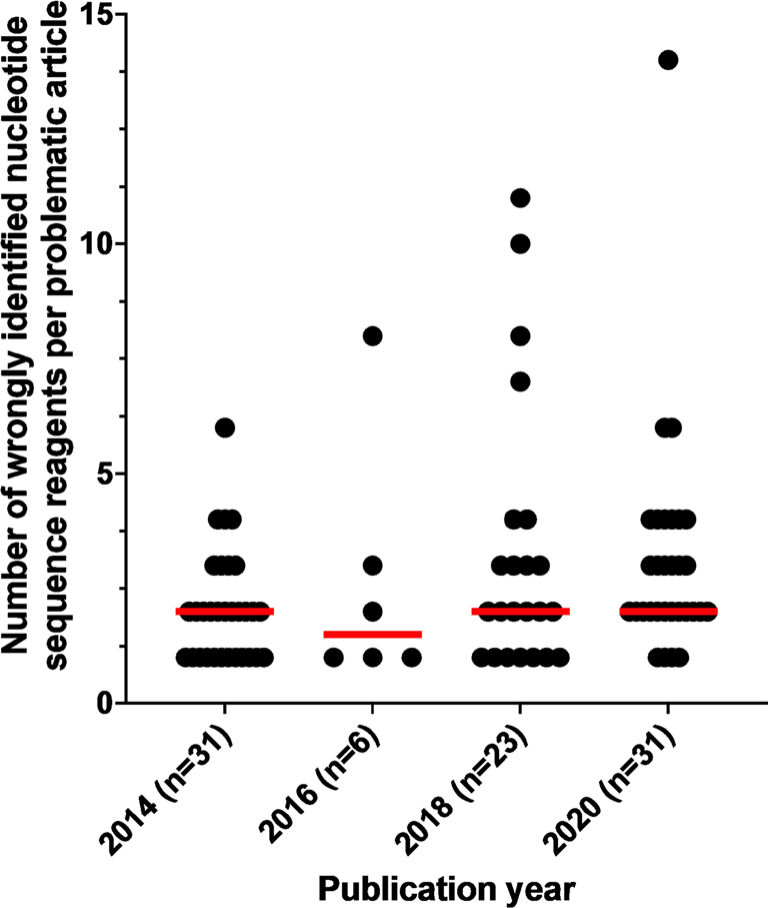
Numbers of wrongly identified nucleotide sequence reagents in *Molecular Cancer* papers (*Y*-axis) according to publication year (*X*-axis). Individual/median numbers of wrongly identified nucleotide sequences/paper are shown as black dots/red horizontal lines, respectively. Numbers of *Molecular Cancer* papers with wrongly identified nucleotide sequence reagent(s) per publication year are shown below the *X*-axis

The 251 wrongly identified nucleotide sequences were distributed across 91/334 (27%) screened *Molecular Cancer* papers (Fig. 5C) and 91/500 (18%) original *Molecular Cancer* papers (Table 2, Fig. 5D, Table S2). These 91 papers included 3 *Molecular Cancer* papers from 2014 that had been previously reported to describe wrongly identified nucleotide sequence(s) (Labbé et al. 2019; Park et al. 2022). Proportions of papers with wrongly identified nucleotide sequence(s) ranged from 6/59 (10%) in 2016 to 31/82 (38%) in 2020 (Fig. 5D). The median number of wrongly identified sequences/paper was 2 (range 1–14) (Table 2, Fig. 6). The numbers of wrongly identified and analyzed sequences per paper were not significantly correlated (Spearman’s rho = 0.1893, 95% Cl =  − 0.02346–0.3857, *p* = 0.0723, *n* = 91).

The 91 *Molecular Cancer* papers with wrongly identified sequence(s) described experiments in human cancer models corresponding to 26 cancer types, most frequently gastric, colorectal, or non-small-cell lung cancer (Table S2). Almost all (84/91, 92%) papers analyzed a single cancer type. One quarter (23/91) of papers with wrongly identified sequence(s) either referred to a specific drug or to chemosensitivity or -resistance in their title (Table S2).

*Molecular Cancer* papers with wrongly identified sequence(s) described a median of 2 genes or transcripts in their titles (range 0–7) (Table S2). Most publication titles (78/91, 86%) mentioned at least one protein-coding gene, and approximately half (48/91, 53%) mentioned non-coding RNA(s) (ncRNAs), which were typically miR(s) (31/48, 65%) or circRNA(s) (15/48, 31%). Whereas most 2014 titles mentioned only protein-coding gene(s) (22/31, 71%), most 2020 titles combined protein-coding gene(s) and ncRNA(s) (22/31, 71%), which were again typically miR(s) (12/22, 55%). Fifteen papers with wrongly identified sequence(s) that referred to circRNA(s) in their titles were published in 2018 and 2020, where titles typically combined circRNA(s) with protein-coding gene(s) and/or miR(s) (13/15, 87%) (Table S2).

### Wrongly identified or non-verifiable reagents for the analysis of human circRNAs

Nine *Molecular Cancer* papers described 20 wrongly identified reagents that were claimed to target circRNAs (Table 3, Table S1). These claimed circRNA targeting reagents were predicted to either target different human transcripts from those claimed (17/20, 85%) or to be non-targeting in human (3/20, 15%) (Table 3). Wrongly identified circRNA targeting sequences included claimed divergent RT-PCR primers that were predicted to amplify linear transcripts, and single reagents that showed significant identity to linear transcripts (see the “Methods” section, Table 3, Table S1). The identities of a further 29 circRNA targeting reagents could not be verified (Table 3), either because the claimed circRNA sequence could not be identified in external databases, or in the case of single reagents, because the BSJ sequence was not provided or identifiable elsewhere (see Methods, Tables S3-S5). Non-verifiable circRNA targeting reagents were identified in 3 *Molecular Cancer* papers that described wrongly identified nucleotide sequence(s) (Tables S3, S5). An additional 6 *Molecular Cancer* papers included non-verifiable circRNA targeting reagents, where all other nucleotide sequences appeared to be correctly identified (Tables S4, S5).

**Table 3:** Wrongly identified and non-verifiable nucleotide sequence reagents that were claimed to target human circRNAs in *Molecular Cancer* and *Oncogene* papers

Claimed circRNA targeting reagents	*Molecular Cancer*	*Oncogene*
Wrongly identified human circRNA targeting reagents	20/20 (100%)	6/6 (100%)
circRNA targeting reagents predicted to target a different human transcript	17/20 (85%)	2/6 (33%)
circRNA targeting reagents that do not discriminate circular and linear isoforms from the claimed host gene	9/17 (53%)	2/2 (100%)
circRNA targeting reagents predicted to be non-targeting in human	3/20 (15%)	4/6 (67%)
circRNA targeting reagents that could not be verified	29/29 (100%)	8/8 (100%)
Claimed circRNA sequence could not be identified in external databases	19/29 (66%)	2/8 (25%)
BSJ sequence for claimed circRNA was not provided or identifiable	10/29 (34%)	6/8 (75%)

### Targeted *Oncogene* corpus

To investigate whether original papers with wrongly identified or non-verifiable nucleotide sequences can be identified in other high IF cancer research journals, we verified nucleotide sequence reagent identities in a subset of original *Oncogene* papers. As described in the Methods, we employed keyword-driven searches of *Oncogene* papers published in 2020, using keywords identified in some *Molecular Cancer* papers (miRNA, miR, circular RNA, or circRNA). This search strategy identified a corpus of 52 *Oncogene* papers that commonly described the analysis of one or more miR’s and/or circRNAs (Table 2). Most (42/52, 81%) selected *Oncogene* papers described human research and at least one nucleotide sequence that was claimed to target a non-modified human gene or genomic sequence. These 42 papers described a median number of 20 sequences/paper (range 2–115) (Table 2).

### *Oncogene* papers with wrongly identified nucleotide sequence(s)

The 42 *Oncogene* papers included 1165 nucleotide sequences, of which 47 (4.0%) sequences were predicted to be wrongly identified (Table 2, Table S1). These 47 wrongly identified sequences were distributed across 21/52 (40%) corpus papers and 21/42 (50%) screened papers (Table S2). These 21 *Oncogene* papers described a median of 2 wrongly identified sequences/paper (range 1–5) (Table 2). *Oncogene* papers with wrongly identified sequence(s) described experiments in human cancer models that corresponded to 14 different cancer types, most frequently breast cancer and hepatocellular carcinoma (Table S2) and referred to a median of 3 genes or transcripts in their titles (range 0–4), where most titles referred to miR(s) (13/21, 62%) (Table S2). Two *Oncogene* papers referred to chemical compounds in their titles (Table S2).

Wrongly identified sequences in 2020 *Oncogene* papers represented targeting reagents that were verified to target a different human gene or genomic sequence from that claimed (24/47, 51%), or claimed targeting reagents that were predicted to be non-targeting in human (23/47, 49%) (Table 2). Six wrongly identified sequences were claimed to target human circRNAs, which were either predicted to be non-targeting in human or to target linear transcript(s) from the claimed host gene (Table 3). A further 8 circRNA targeting sequences were not verifiable, either because the relevant BSJ sequence was not provided or because the claimed circRNA sequence could not be identified (Table 3, Tables S3, S5).

### Countries of origin and institutional affiliations of *Molecular Cancer* and *Oncogene* papers with wrongly identified nucleotide sequence(s)

*Molecular Cancer* and *Oncogene* papers with wrongly identified sequence(s) were authored by teams from 12 and 5 different countries, respectively (Table 4, Table S2). Most *Molecular Cancer* (67/91, 74%) and *Oncogene* papers (17/21, 81%) were authored by teams from China, followed by authors from USA in the case of *Molecular Cancer* (7/91, 8%) (Table 4). When papers with wrongly identified sequence(s) were analyzed according to both country and institution of origin (Park et al. 2022), most *Molecular Cancer* and *Oncogene* papers from China were affiliated with hospitals, compared with minorities of papers from other countries (Table 4). Significantly more *Molecular Cancer* papers from China were authored by hospital-affiliated teams (57/67 (85%)), compared with papers from other countries (6/24 (25%)) (Fisher’s exact test, *p* < 0.0001, *n* = 91) (Table 4).

**Table 4:** *Molecular Cancer* and *Oncogene* papers with wrongly identified nucleotide sequence reagent(s) according to country of origin and institutional affiliation type

	*Molecular Cancer*			*Oncogene*		
Country of origin	All problematic papers	Hospital affiliated	Not hospital affiliated	All problematic papers	Hospital affiliated	Not hospital affiliated
All countries	91 (100%)	63/91 (70%)	28/91 (30%)	21 (100%)	12/21 (57%)	9/21 (43%)
China	67/91 (74%)	57/67 (85%)	10/68 (15%)	17/21 (81%)	12/17 (70%)	5/17 (30%)
All other countries	24/91 (26%)	6/24 (25%)	18/24 (75%)	4/21 (19%)	0/4 (0%)	4/4 (100%)
* USA*	7/91 (8%)	0/7	7/7	–	–	–
* Germany*	4/91 (4%)	2/4	2/4	–	–	–
* France*	3/91 (3%)	1/3	2/3	–	–	–
* India*	2/91 (2%)	0/2	2/2	–	–	–
* Spain*	2/91 (2%)	1/2	1/2	–	–	–
* Italy*	1/91 (1%)	0/1	1/1	–	–	–
* Japan*	1/91 (1%)	0/1	1/1	1/21 (5%)	0/1	1/1
* Singapore*	1/91 (1%)	0/1	1/1	–	–	–
* Taiwan*	1/91 (1%)	0/1	1/1	1/21 (5%)	0/1	1/1
* Thailand*	1/91 (1%)	0/1	1/1	–	–	–
* UK*	1/91 (1%)	0/1	1/1	1/21 (5%)	0/1	1/1
* Belgium*	–	–	–	1/21 (5%)	0/1	1/1

### Citations and post-publication commentary/corrections of *Molecular Cancer* and *Oncogene* papers with wrongly identified nucleotide sequence(s)

**Fig. 7:**
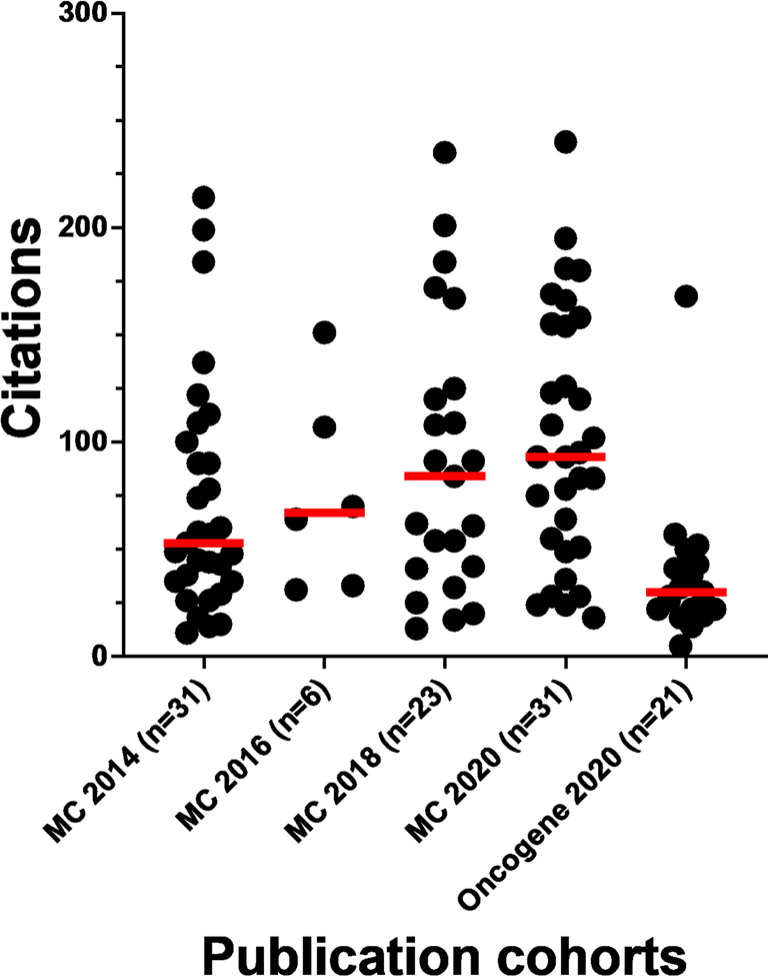
Google Scholar citations of *Molecular Cancer* and *Oncogene* papers with wrongly identified nucleotide sequence reagent(s) (*Y*-axis) according to journal and publication year (*X*-axis). Individual/median citation numbers are shown as black dots/red horizontal lines, respectively. Numbers of *Molecular Cancer* (MC) or *Oncogene* papers per year are shown below the *X*-axis

The 91 *Molecular Cancer* papers with wrongly identified nucleotide sequence(s) have been collectively cited 7932 times according to Google Scholar (Table S2). Some 33 *Molecular Cancer* papers have been cited at least 100 times, and 27 others have been cited at least 50 times (Fig. 7). Highly cited papers include 22 papers published in 2020 (Fig. 7). The 21 *Oncogene* papers from 2020 have been cited 878 times according to Google Scholar (Table S2), where one paper has been cited 168 times, and 5 other papers have been cited at least 50 times (Fig. 7).

Ten *Molecular Cancer* papers and 4 *Oncogene* papers with wrongly identified nucleotide sequence(s), and one *Molecular Cancer* paper with non-verifiable circRNA targeting reagents have associated published corrections, mostly in response to concerns about image integrity (Table 5). Two *Molecular Cancer* papers were corrected for wrongly identified sequences (Table S6), where one paper had been previously identified by our team (Park et al. 2022). In the other published correction, one nucleotide sequence remained wrongly identified in the correction notice (Table S6). Four *Molecular Cancer* papers have been retracted in response to image integrity and ethics concerns (Table 5). Just under one third (26/91, 29%) of *Molecular Cancer* papers and 5/21 (24%) *Oncogene* papers have been flagged on PubPeer, mostly for image integrity concerns (Table 5). Four *Molecular Cancer* papers have been flagged on PubPeer for wrongly identified nucleotide sequences, including one paper from a previous study (Labbé et al. 2019) (Table 5).

**Table 5:** Post-publication notices and PubPeer commentary for *Molecular Cancer* and *Oncogene* papers

	*Molecular Cancer*	*Oncogene*
**Problematic papers with wrongly identified nucleotide sequence(s) (proportion (percentage))**	**91/91 (100%)**	**21/21 (100%)**
Published corrections*	**10/91 (11%)**	**4/21 (19%)**
Images	9/10 (90%)	3/4 (75%)
Wrongly identified nucleotide sequences**	2/10 (20%)	–
Typographical errors	1/10 (10%)	–
Statistics	–	1/4 (25%)
Retractions*	**4/91 (4%)**	–
Images	4/4 (100%)	–
Ethics	2/4 (50%)	–
Flagged on PubPeer*	**26/91 (29%)**	**5/21 (24%)**
Images	23/26 (89%)	4/5 (80%)
Wrongly identified nucleotide sequences**	4/26 (15%)	–
Methodology	2/26 (7%)	2/5 (40%)
Ethics	1/26 (4%)	–
Concerns about published correction	1/26 (4%)	–
Duplicate publication	1/26 (4%)	–
**Papers describing non-verifiable circRNA targeting reagent(s)**	**6/6 (100%)**	–
**Published corrections**	**1/6 (17%)**	–
Typographical error (circRNA identifier)	1/1 (100%)	–

^*^Some papers were corrected/retracted/flagged on PubPeer due to multiple issues

^**^Papers corrected or flagged on PubPeer because of wrongly identified sequences include 3 previously reported *Molecular Cancer* papers

## Discussion

Verifying the identities of nucleotide sequences published in *Molecular Cancer* has shown that 10–38% of all original *Molecular Cancer* papers published in 2014, 2016, 2018, and 2020 papers described wrongly identified nucleotide sequence(s). These proportions also rose from 2014–2020, when the journal IF increased from 4.3 to 27.4 (Fig. 1). We identified similar papers in the journal *Oncogene*, where 40% papers published in 2020 that studied miRs and/or circRNAs were found to describe wrongly identified nucleotide sequence(s). Many of these *Molecular Cancer* and *Oncogene* papers have been highly cited, including publications from 2020. These results support and extend previous findings demonstrating that human gene research papers with wrongly identified nucleotide sequences can be identified in high IF journals (Labbé et al. 2019; Park et al. 2022).

The analysis of *Molecular Cancer* and *Oncogene* papers that examined circRNAs in human cancer also identified incorrect circRNA targeting reagents, where some errors reflected the particular requirements of circRNA targeting reagents (Dudekula et al. 2016; Zhong et al. 2018; Nielsen et al. 2022). As also reported by Zhong et al. (2019), we identified claimed divergent RT-PCR primers that did not appear to discriminate between circular and linear transcripts, as well as single reagents that did not appear to be specific for the claimed circRNA target. The identities of other circRNA targeting reagents could not be verified, either because the claimed circRNA sequence or the BSJ sequence was not provided and/or could not be identified elsewhere. These results add to previous descriptions of cancer research papers in which claimed circRNAs could not be independently verified (Patop and Kadener 2018).

### Study limitations

Before discussing our results further, it is important to recognize our study’s limitations, as well as study design factors that may have identified higher proportions of papers with wrongly identified nucleotide sequence reagent(s) than those previously reported (Park et al. 2022) (Table 6). We recognize that the present study has examined original papers from only two journals, due to the challenges of manually verifying nucleotide sequence identities in papers that frequently described 50–100 sequences per paper. In previous studies, we employed the semi-automated Seek & Blastn tool (Labbé et al. 2019), which screens publications for short nucleotide sequences and then verifies their claimed identities using blastn (Altschul et al. 1990). Screening original papers with Seek & Blastn and then manually verifying the results found that up to 4.2% and 12.6% of 2014–2018 papers in the journals *Gene* and *Oncology Reports* described wrongly identified nucleotide sequence(s) (Park et al. 2022). In the present study, every *Molecular Cancer* and *Oncogene* paper was analyzed manually, which may have reduced false-negative results associated with Seek & Blastn screening (Labbé et al. 2019; Park et al. 2022) (Table 6). At the same time, manual verification of nucleotide sequence identities does not preclude the possibility of human errors leading to false-positive results, particularly where thousands of individual nucleotide sequences are analyzed (Table 6).

**Table 6:** Strengths and weaknesses of manual validation of nucleotide sequence reagent identities

Strengths	Weaknesses or limitations
• Manual nucleotide sequence extraction potentially associated with fewer skipped or wrongly extracted nucleotide sequence reagents • Facilitates analysis of reagents with different targeting requirements (e.g., reverse RT-PCR primers for miRNA targets, circRNA targeting reagents) • Facilitates identification of new or unexpected error types • Facilitates analysis of nucleotide sequence reagents that are claimed to target different species	• Laborious • Time-consuming • Difficult to scale, particularly where publications describe many individual nucleotide sequence reagents • Susceptible to operator error through fatigue • Intensive use of online resources such as Blastn can result in users being blocked (e.g., recognized as suspicious activity)

The numbers of nucleotide sequences per *Molecular Cancer* paper also rose significantly from 2014 to 2020 (Fig. 4B). It seems possible that as the numbers of nucleotide sequence reagents per paper increase, more papers could describe wrongly identified sequences. However, we noted that the median numbers of wrongly identified sequences per *Molecular Cancer* paper were largely stable across 2014–2020, and no significant correlation was measured between wrongly identified and overall nucleotide sequence numbers. Median numbers of wrongly identified sequences in *Molecular Cancer* and *Oncogene* papers were also similar to those noted for papers in lower IF journals (Park et al. 2022). This suggests that the rising proportions of erroneous *Molecular Cancer* papers from 2014 to 2020 do not simply reflect the publication of increasingly complex papers during this time.

### Possible explanations for wrongly identified nucleotide sequences

Wrongly identified nucleotide sequences can clearly occur in the context of genuine research (Park et al. 2022), particularly where papers describe many individual reagents (Table 1). At the same time, many nucleotide sequence identity errors in *Molecular Cancer* and *Oncogene* papers seem inconsistent with errors that might be made by expert authors, such as claimed human gene targeting sequences with no identifiable human target, where some sequences were instead predicted to target orthologous genes in species other than human. As we have previously described, research experts seem unlikely to select human gene targeting reagents that do not target any human gene (Park et al. 2022). Most researchers will also be aware that nucleotide sequence reagents that are identical to gene sequences in rodents, plants, or fungi will be unlikely to effectively target the orthologous human gene (Park et al. 2022). We were also surprised to discover numerous claimed circRNA targeting siRNAs that did not appear to target the claimed BSJ, despite the BSJ sequence being provided by the authors.

We recognize that as an external research team, we cannot draw firm conclusions about significance of the nucleotide sequence errors that we have described, or the contexts in which these errors occurred. Nonetheless, numerous papers in *Molecular Cancer* and *Oncogene* with wrongly identified nucleotide sequences could support other journals’ concerns that paper mills may be successfully targeting some high IF journals (Heck et al. 2021; Bricker-Anthony and Giangrande 2022; Frederickson and Herzog 2022). Given the prestige associated with publishing in high IF journals, some paper mills and clients could value or require publications in high IF journals, which may become acute as lower IF journals are recognized as possible paper mill targets (Zhang et al. 2022b). As the price per paper mill manuscript may be partly dictated by journal IF (Abalkina 2023), publishing in high IF journals could allow paper mills to charge higher manuscript fees, which could allow paper mills to produce more sophisticated manuscripts that more closely resemble genuine papers. Developments in artificial intelligence, in terms of both text (Floridi and Chiriatti 2020; Grimaldi and Ehrler 2023) and image generation (Wang et al. 2022; Gu et al. 2022), could add to paper mill capacity to produce sophisticated manuscripts that could meet the expectations of some high IF journals.

### Impact of wrongly identified reagents in high IF journals

Due to limitations in available time and human cognition, academics and researchers have consistently described reading between ~ 150 and 400 research publications per year (Tenopir et al. 2009, 2015, 2019). As these numbers of papers are greatly exceeded by the quantity of available literature, many researchers use heuristics to help decide which papers they should read (Tenopir et al. 2016; Nicholas et al. 2019; Morales et al. 2021; Teplitskiy et al. 2022). Survey results consistently report that academics and researchers prioritize reading papers in high IF journals and/or with high citation numbers (Tenopir et al. 2016; Nicholas et al. 2019; Teplitskiy et al. 2022), where early career researchers may place more emphasis on journal IF and citations as proxies for research quality (Tenopir et al. 2016; Nicholas et al. 2019).

The repeated demonstration of researcher preferences for papers in high IF journals (Tenopir et al. 2016; Nicholas et al. 2019; Teplitskiy et al. 2022) means that publications in high IF cancer journals that describe wrongly identified nucleotide sequence reagents could impact future research. Highly cited papers in high IF journals are likely to be prioritized for reading (Tenopir et al. 2016; Nicholas et al. 2019; Teplitskiy et al. 2022), where a proportion of these papers could be used in future research. Researchers may also be more motivated to reproduce results published in high IF journals, as reflected by the design of the Cancer Biology Reproducibility Project that attempted to reproduce cancer research studies published in high IF journals (Errington et al. 2021). Gene research papers in high IF cancer journals could therefore encourage more researchers to attempt new research, and potentially waste time and resources through the experimental use of wrongly identified reagents (Park et al. 2022; Byrne et al. 2022). In cases where papers with wrongly identified reagents describe significant associations between gene expression and drug sensitivity or resistance, they could also stimulate potentially futile research in adjacent research fields such as pharmacology.

Due to the direct relationship between citation numbers and journal IF, citations to papers with wrongly identified nucleotide sequences could also be generating a positive feed-forward loop within the human gene literature. Highly cited gene research papers can boost journal IF, which could then bring these papers to the attention of more researchers who use journal IF and citation numbers as proxies for research quality (Tenopir et al. 2016; Nicholas et al. 2019). Awareness that ncRNA papers can attract high citation numbers (Fire and Guestrin 2019) could also encourage a range of journals to consider manuscripts that describe ncRNA research. The confluence between citation potential of ncRNA publications (Fire and Guestrin 2019) and the possible value of these gene topics to paper mills (Byrne and Christopher 2020; Cooper and Han 2021; Park et al. 2022; Pérez-Neri et al. 2022; Byrne et al. 2022; Wittau et al. 2023) could lead to the unintended acceptance of problematic human gene research manuscripts by high IF journals, which could then bring these publications to the attention of more researchers.

### Suggested next steps

The identification of papers with wrongly identified nucleotide sequence reagents in high IF cancer research journals should encourage the analysis of recent papers in other high IF journals, including journals that publish gene research of relevance to pharmacology. Problematic papers in high IF journals could demonstrate the leading edge of paper mill capability and could help to predict the types of manuscripts that could be received by a broader range of journals in future (Byrne et al. 2022). The possibility of paper mills harnessing new and rapidly developing capacities for automated text generation (Grimaldi and Ehrler 2023) highlights the urgent need for more critical analyses of papers in high IF journals.

The field of circRNA research is also growing rapidly, where the majority of circRNA papers have been published by authors from few countries (Wu et al. 2021; Zhang et al. 2022a). In light of our results, we speculate that laboratory research involving circRNAs may be vulnerable to exploitation by paper mills. Incomplete and non-overlapping circRNA databases that can include poorly or incompletely annotated circRNA sequences (Costa and Enguita 2020; Dodbele et al. 2021; Vromman et al. 2021), combined with multiple circRNA nomenclature systems (Costa and Enguita 2020; Dodbele et al. 2021; Vromman et al. 2021; Nielsen et al. 2022), can collectively underpin superficial published descriptions of individual circRNAs, and render poor-quality circRNA research more challenging to detect. Individual circRNAs can also be linked with many different protein-coding genes and ncRNAs (Kristensen et al. 2018; Dodbele et al. 2021), which could enable the creation of large numbers of manuscripts that combine different circRNAs, ncRNAs, protein-coding genes, and/or drug treatments across different diseases such as human cancer types. The rapid growth in the numbers of circRNA papers (Dodbele et al. 2021; Wu et al. 2021; Zhang et al. 2022a) could also limit the availability of expert peer reviewers with in-depth knowledge of critical factors in circRNA research.

Our analyses show that some human circRNA papers in high IF journals are setting poor standards for methods and results reporting, particularly for readers who may be unfamiliar with the requirements of circRNA targeting reagents. Some descriptions of circRNA research in *Molecular Cancer* and *Oncogene* indicate the need for better reporting of circRNAs and their targeting reagents (Table 7), as also recognized by others (Kristensen et al. 2018; Patop and Kadener 2018; Costa and Enguita 2020; Dodbele et al. 2021; Vromman et al. 2021; Nielsen et al. 2022). The poor reporting practices that we and others have identified (Table 7) indicate the need for specific guidance around circRNA (reagent) reporting, and for such guidance to be more strictly enforced. Journals and publishers can take further steps to promote full disclosure and accurate reporting of nucleotide sequence reagents (Table 8), where high IF journals are well placed to show leadership on best practices.

**Table 7:** Recommendations for improved reporting of circRNA sequences and circRNA targeting reagents in research publications

Problems	Proposed solutions
• Non-verifiable circRNA targeting reagents as claimed circRNA and/or sequence could not be identified (Patop and Kadener 2018) • Identities of claimed circRNA targets unclear or poorly described (Vromman et al. 2021) • Case sensitive circRNA identifiers	• circRNA sequences to be described in publications and deposited in external databases with clearly disclosed accession information (Dodbele et al. 2021; Vromman et al. 2021) • circRNA sequence descriptions to specify whether sequence is complete or partial • circRNAs to be identified by unique, recognized identifiers that disclose the host gene (Kristensen et al. 2018; Costa and Enguita 2020; Vromman et al. 2021) • circRNA identifiers to be accompanied by circRNA genomic coordinates, including reference genome build (Costa and Enguita 2020; Nielsen et al. 2022) • circRNA identifiers to be linked to database entries in publications • circRNA database search algorithms to accept case-insensitive circRNA identifiers as queries
• circRNA reagents could not be verified as BSJ sequence not provided or traceable (Vromman et al. 2021) • Limited BSJ sequence information provided in publications • BSJ sequences shown in images, ie flat files, not machine readable	• circRNA descriptions to include transparent and verifiable information about BSJ sequence (Dodbele et al. 2021; Vromman et al. 2021) • circRNA databases to annotate BSJ at sequence level and define whether BSJ is predicted or experimentally verified • Published BSJ sequences to disclose at least 5–16 nts on each side of BSJ • BSJ sequences to be written in machine-readable format, preferably within the main text of publication
• Unclear targeting parameters for single circRNA targeting reagents • circRNA targeting RT-PCR primers not specified as divergent or convergent	• Single circRNA reagent descriptions to specify whether reagent targets the BSJ, or other circRNA feature not conserved in linear transcripts • circRNA reagent descriptions to specify intended experimental use, including all reagents described in supplementary tables/files

**Table 8:** Recommended actions to improve the reporting of nucleotide sequence reagents

Recommended actions	Reference(s)
All nucleotide sequence reagents to be fully disclosed and written in correct orientation (5′-3′)	Chiarella et al. (2015) Bustin and Nolan (2017)
Nucleotide sequence reagents to be published in machine readable formats without internal spacing or line, column, or page breaks	Labbé et al. (2019)
Each nucleotide sequence reagent to be linked with intended target and experimental purpose: • Reagent identities to be immediately adjacent to relevant sequences within text and/or tables • Targeting information to refer to universally-recognized gene/transcript/genomic sequence nomenclature and targeted species • Genomic coordinates of targeting reagents to include genomic build information, where relevant	Chiarella et al. (2015) Labbé et al. (2019)
Authors to include statements confirming that all nucleotide sequence reagent identities have been checked and are correct to authors’ best knowledge	Labbé et al. (2019)
All publications with wrongly identified nucleotide sequence(s) to be rapidly and transparently flagged through editorial notes or expressions of concern	Byrne and Christopher (2020)
Journals and publishers to proactively identify and flag all papers with known wrongly identified reagents, such as incorrect non-targeting controls	
Where wrongly-identified nucleotide sequence reagents are not promptly addressed by authors, publications to be considered for retraction	

## Summary and conclusions

Despite well-recognized limitations in the use of journal IF to predict research quality (Ioannidis and Thombs 2019; Siler and Larivière 2022), high IF journals are valued and relied upon by many biomedical researchers. Our results indicate that contrary to reasonable expectations, gene research papers with wrongly identified nucleotide sequence reagents may be frequent in some high IF cancer journals. This highlights the need for biomedical researchers to exercise caution when interpreting published gene research, including research published in high IF journals. Publications must not be exempt from critical analysis simply because they have been published in a high IF journal and/or achieved seemingly impressive numbers of citations. These findings also support recommendations that trainee and researcher education programs actively discuss features of trustworthy publications (Byrne et al. 2022).

Misplaced beliefs that paper mills are only a problem for lower IF journals risk exacerbating the vulnerability of high IF journals towards paper mills. Given their established brands, reputations, and available resources, we hope that high IF journals and their publishers will be responsive to reports of gene research papers with verifiable reagent errors and will lead efforts in recognizing and responding to threats posed by research paper mills.

### Supplementary Information

Below is the link to the electronic supplementary material.

Supplementary file1 (XLSX 61 KB)

## Data Availability

All data generated or analyzed during this study are included in this published article and its Supplementary Information files. All information extracted from or about analyzed publications, as well as Google Scholar citation data and PubPeer notifications is available within the public domain.
