# Publisher Correction: Verification of nucleotide sequence reagent identities in original publications in high impact factor cancer research journals

**DOI:** 10.1007/s00210-024-02953-8

**Published:** 2024-01-16

**Authors:** Pranujan Pathmendra, Yasunori Park, Francisco J. Enguita, Jennifer A. Byrne

**Affiliations:** 1 School of Medical Sciences, Faculty of Medicine and Health, The University of Sydney, Camperdown, NSW 2050 Australia; 2 Instituto de Medicina Molecular João Lobo Antunes, Faculdade de Medicina, Universidade de Lisboa, Av. Prof. Egas Moniz, 1649‑028 Lisbon, Portugal; 3 NSW Health Statewide Biobank, NSW Health Pathology, Camperdown, NSW 2050 Australia

**Publisher Correction: Naunyn-Schmiedeberg's Archives of Pharmacology**

10.1007/s00210-023-02846-2

After the publication of the original article, the author noticed that the image of Fig. 1 has been replaced with a different image that is not relevant to their paper. The error originated during the final stage of the publication process. The publisher sincerely apologies for this mistake and the inconvenience caused and assumes responsibility for this mistake.

The correct image is shown below.

**Fig. 1:**
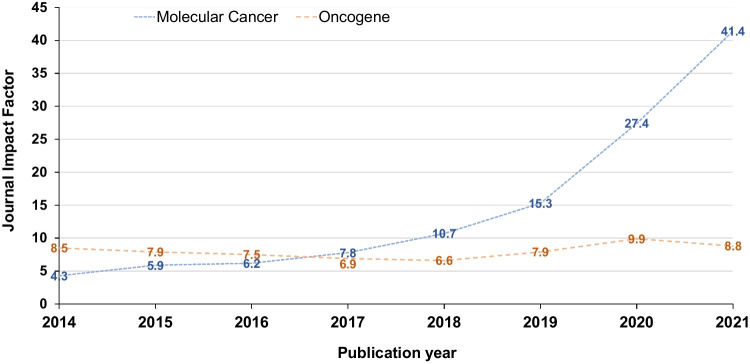
Journal impact factors (https://clarivate.com/) (*Y*-axis) for *Molecular Cancer* (blue) and *Oncogene* (orange) from 2014 to 2021 (X-axis). Journal impact factors have been rounded to one decimal place

The original article has been corrected.

